# Self-Employment Transitions and Health Outcomes in Later Life: Evidence From China

**DOI:** 10.1093/geroni/igae073

**Published:** 2024-08-11

**Authors:** Ting Hu, Yu-Chih Chen, Cal Halvorsen

**Affiliations:** School of Social Work, University of Illinois at Urbana-Champaign, Urbana, Illinois, USA; Department of Social Work and Social Administration, The University of Hong Kong, Hong KongChina; Social Policy Institute, Washington University in St. Louis, St. Louis, Missouri, USA; Brown School of Social Work, Washington University in St. Louis, St. Louis, Missouri, USA; Center for Work, Health, & Well-being, T.H. Chan School of Public Health, Harvard University, Cambridge, Massachusetts, USA

**Keywords:** Cognitive function, Depressive symptoms, Productive engagement, Satisfaction, Work

## Abstract

**Background and Objectives:**

Self-employment is a vital alternative to waged employment for older workers. Recent research has shown that employment transitions frequently occur when individuals approach retirement. However, evidence of how older people’s health changes when they switch between self and waged employment is lacking, particularly outside Western contexts. To address this research gap, we explored the health impact of employment transitions for the older working population in China by hukou (urban or rural household registration status), region, and education.

**Research Design and Methods:**

We employed fixed effect models to examine the impact of employment transitions on cognitive, mental, and physical health and life satisfaction drawing on data from four waves (2011, 2013, 2015, and 2018) of the China Health and Retirement Longitudinal Study (*N* = 4,606). Given China’s unique context, we analyzed the results of agricultural and nonagricultural work separately.

**Results:**

Individuals transitioning into or remaining in self-employment had lower self-rated health and life satisfaction than those remaining in waged employment. There was no significant difference in cognitive functioning or depressive symptoms. Additionally, those who transitioned from self-employment into waged employment rated their health worse than those who remained in waged employment. The health impacts were more apparent for agricultural than nonagricultural self-employment, particularly for older workers living in urban regions with rural hukou and lower education levels.

**Discussion and Implications:**

Most older Chinese transitioning into or staying self-employed are or were pushed into self-employment due to their low human capital and socioeconomic status, which affects their subsequent health. Pension reform and policies supporting older adults to stay in the workforce could help close the economic and health gaps between rural and urban older adults.

**Translational Significance:** Employment transitions frequently occur when one approaches retirement. Self-employment is an alternative to waged employment for workers facing retirement. Examining the employment transitions in the Chinese context, this study shows that those transitioning to or staying self-employed reported lower self-rated health and life satisfaction. However, those with socioeconomic advantages reported better cognition and self-rated health. This study highlights that policies should provide economic and health support to older workers, especially those with socioeconomic disadvantages. Policy measures, such as pension reform and skill training, should be implemented to promote longer and healthier working lives.

Employment is an important social determinant of health over the life course (Lovejoy et al., 2021). However, as retirement approaches, individuals often experience employment transitions that involve switching jobs or careers and moving in and out of retirement (Owen & Flynn, 2004). Rather than immediately leaving the labor force, individuals may opt for flexible employment to stay in the labor market, in which they may change from full-time to part-time employment or switch types of employment between waged or salaried (hereafter “waged employment”) and self-employment (Giandrea et al., 2008).

Self-employment is usually defined as working for oneself, as opposed to waged employment that works for other people or organizations (Halvorsen, 2021; Zissimopoulos & Karoly, 2007). It is a flexible and catch-all employment type that includes small business owners, entrepreneurs (Pitt-Catsouphes et al., 2017), freelancers, and independent contractors (Weller et al., 2015). It can also include the incorporation of a new business. Research has shown that self-employment is a crucial alternative to waged employment and is associated with cognitive, mental, and physical health (Ahn, 2020; Chanda & Banerjee, 2022; Nikolova, 2019; Stephan et al., 2020). Yet the context of the country matters, with self-employment rates varying extensively among older workers between 32 countries within the Organization for Economic Co-Operation and Development (Gerpott et al., 2021).

Previous studies on the older population, predominately in Western and high-income societies, have examined transitions between retirement and work (Justo et al., 2021; Solinge, 2014). However, there has been limited investigation of movements between waged and self-employment elsewhere and how such transitions may affect health. With an increasing number of older adults entering the workforce, understanding whether and how employment transitions affect health is crucial, as it can inform policies and interventions to promote longer working lives and healthy aging. This study addresses this critical gap by examining the health impacts of transitions between waged and self-employment among older workers in China.

## Employment Transitions in Later Life

Employment transitions generally comprise two types: transitions between employment types (e.g., waged employment and self-employment) or transitions between being employed or not (e.g., retirement or others; see Bruce et al., 2000). Previous studies involving older adults have predominately examined transitions between employment and retirement (Justo et al., 2021; Solinge, 2014). However, late-career transitions between employment types also occur (Christelis & Fonseca, 2015; Hipple, 2004, 2010).

Push or pull factors may drive individuals to choose self-employment (Halvorsen & Morrow-Howell, 2017). Individuals may be pushed into self-employment to avoid unemployment or underemployment, particularly among those with lower human (e.g., poor health or lower education) or financial capital (e.g., low income or wealth) (Block & Wagner, 2010). Conversely, individuals with better skills, experience, or more social and financial resources may be drawn to self-employment to pursue greater autonomy and flexibility (Nikolova, 2019; Stephan et al., 2020).

## Theoretical Framework

The impact of employment transitions on health and mental well-being can be framed by the job demand-control model (Karasek, 1979), which suggests that an individual’s response to work-related stress can either increase or mitigate the effects of job demands and control on their stress levels. This model posits that job stress is a function of two primary factors: job demands and job control. Job demands refer to physical and/or psychological workload and time pressure associated with a specific job, which can lead to increased job stress. Conversely, job control refers to the degree of autonomy and decision-making power an individual has over work tasks. An expanded version of this model incorporating massive technological advances, globalization, and employer practices suggests how jobs can be designed to promote better health and well-being (Lovejoy et al., 2021). For example, jobs that provide more schedule control, worker input, and voice may lead to better employee outcomes. Further, the expanded model highlights the positive impact that social support and social networks have on employees. As such, the interplay between job demands, control, and social support may have varying impacts on older workers’ health and well-being.

Applying the job demand-control model to self-employment leads to a plausible hypothesis that self-employment may both positively and negatively affect health and well-being outcomes. On the one hand, self-employment can offer greater autonomy and control over work tasks, leading to reduced job demands and lower levels of stress, thereby improving health outcomes such as reduced blood pressure, lower levels of anxiety and depression, and better overall mental health (Bencsik & Chuluun, 2021; Nikolova, 2019). On the other hand, being self-employed may entail higher job demands due to managing multiple tasks and responsibilities and the expectation of working longer hours (Ahn, 2020; Lewin-Epstein & Yuchtman-Yaar, 1991). Self-employed workers may experience lower levels of job control because they have to manage all aspects of their business, such as finances and administration, potentially increasing stress and adverse health outcomes, particularly if they do not have adequate resources and support to manage these demands.

Aside from occupational characteristics, health status associated with self-employment transitions is also influenced, in part, by individual motivations. Older adults who voluntarily choose self-employment may experience better health outcomes because the flexibility of their daily routines enables them to engage in physical exercise and reduces stress levels by providing greater autonomy and flexibility (Frey & Benz, 2008; Kautonen et al., 2017). In contrast, those forced into self-employment may encounter higher stress levels and poorer working conditions, potentially negating the benefits of self-employment or even worsening health outcomes (Block & Wagner, 2010).

## Self-Employment in China

Studies conducted in Western developed countries generally report positive impacts on mental and life satisfaction of employment transitions for individuals when switching from waged employment to self-employment (Binder & Coad, 2013; Frey & Benz, 2008; Kautonen et al., 2017; Nikolova, 2019). These positive results may also be explained, in part, by the finding that transitioning to self-employment in later life leads to increases in self-realization, such as pursuing self-directed goals and new opportunities (Kautonen et al., 2023). For example, U.S. research has shown that being male, white, married, more highly educated, and having a higher income increases the likelihood of being drawn to self-employment to enjoy the benefits of autonomy and flexibility (Halvorsen, 2021; Weller et al., 2015). However, such a positive impact may not be evident in China, as older adults may be more likely to be pushed into self-employment because of China’s unique household registration system and discrimination against older workers.

In 1958, China established a household registration structure known as the hukou system, which assigns individuals an agricultural or nonagricultural hukou (i.e., urban household registration status) in a particular location based on their parents’ hukou status. The hukou system’s purposes are to protect the interests of Chinese citizens and the legitimacy of living and working in assigned urban or rural areas. Nevertheless, it has established a legal rural–urban dichotomy in addition to the existing practical rural–urban gap and has become a significant contributor to social stratification in China and governs population movement while determining individuals’ access to social welfare (e.g., retirement pension and social insurance), services (e.g., education, healthcare, and housing), and labor market opportunities. As individuals inherited hukou status from their parents, changing hukou status is not permitted unless under certain exceptions (e.g., rural hukou holders can obtain urban hukou status by fulfilling education requirements or being discharged from the military; Wu & Treiman, 2004). The changes in living areas, such as urbanization, do not affect individuals’ hukou status.

This institutional design creates a segregated welfare system in which individuals holding urban hukou enjoy better welfare, such as relatively better economic support and quality of care, than their rural hukou counterparts. This hukou system further interacts with the areas (i.e., rural or urban areas) where individuals reside. For example, the hukou system creates a “*floating population*,” representing the labor workers—typically farmers with rural hukou—who left rural areas seeking work in urban cities as a response to agricultural reforms starting in the early 1980s (Peng, 2018). Individuals with rural hukou could not enjoy the welfare benefits in the urban areas since their welfare entitlement was linked to their registration in a rural location.

Such an institutional arrangement further interacts with retirement age, affecting well-being and health later in life. The mandatory retirement age in China is 60 for men and 50 (blue-collar) to 55 (white-collar) for women. Individuals with urban hukou are expected to retire from formal employment when they reach retirement age and seek a new job if they wish to continue working. These older adults could be re-employed through personal relationships or self-employment, although few obtain a new job through being rehired by their former employer, market recruitment, or government assistance (Peng, 2018). Those with rural hukou, who generally work in businesses collecting or producing natural resources, often must continue working due to insufficient pension and other retirement income (Benjamin et al., 2003).

Regardless of hukou status, older Chinese adults face many challenges in finding a new job because of age discrimination and the lack of job-specific human capital such as education, job skills, and knowledge (Chou, 2010). Lower human capital has generally limited older people’s re-employment choices, pushing most older adults into manual work (Gong et al., 2015). Intensified market competition and ever-changing occupational requirements generate challenges for older adults, such as insufficient technology and management skills, limited job-seeking channels, inadequate competitiveness, and mismatched market demand (Lo, 2023). A recent survey by a Chinese consulting firm reported that 41% of respondents aged 60 or over cited age discrimination as the main reason for being rejected for employment (Nulimaimaiti, 2022). Self-employment, compounded with institutional factors and the pursuit of income maintenance, is considered a choice for older Chinese adults who wish to continue in the labor force.

In summary, although self-employment is generally defined as working for oneself (Halvorsen, 2021; Zissimopoulos & Karoly, 2007), the unique Chinese context adds nuanced attributes to self-employment. Similar to the concept in the Western literature, self-employment in the Chinese context includes various nonagricultural occupations, such as off-farm self-employment involving firm owners, retail shop owners, construction contractors, and lorry owners delivering goods (Gustafsson & Zhang, 2022). However, given that many older workers in China also engage in the agriculture sector (Peng, 2018), self-employment may also include agricultural components involving farmers and small-scale livestock operators (Zhang et al., 2006). In essence, considering both nonagricultural and agricultural aspects of self-employment may be necessary to comprehend fully the characteristics of the labor force for older Chinese.

## The Role of Locality and Education

In addition to hukou status, location, and education may also influence self-employment (Luo & Chong, 2019). Although hukou status influences an *individual’s* eligibility for social welfare and services, location (i.e., rural or urban) influences an entire *region’s* access to employment, health, and social infrastructure, and rural location is associated with fewer of these resources (Lin & Rodgers, 2018; Tani, 2017). The contextual differences between rural and urban areas may affect the opportunities and occupational structure of self-employment. The most available occupations for older adults in rural areas are agricultural-related (e.g., farming, animal husbandry, fishing, and water conservancy) because of policies favoring urban development (Peng, 2018).

Accelerating urbanization and the outflow of young and middle-aged rural farmers make left-behind older adults the key component of the rural permanent population and labor force, the vast majority (88.5%) of whom work in agriculture (Peng, 2018). The remainder are engaged in nonagricultural work, such as construction, handling, processing, and transportation, with working conditions similar to or worse than agricultural work (Zhu & Huang, 2020). However, the occupational structure in urban areas is more diversified, suggesting that the nature and occupational structure of self-employment and the resulting health outcomes may vary between rural and urban areas. Urban self-employment is more diverse, including manufacturing, services, and selling goods (Peng, 2018). In contrast, most rural self-employment involves agricultural work. Therefore, differentiating the nature of self-employment into agricultural and nonagricultural self-employment may enhance understanding of its effect on health and well-being in later life.

Furthermore, education is a crucial driver of motivation for work (Yabiku & Schlabach, 2009). Individuals with varying levels of education may choose self-employment to maintain their post-retirement income (Caballero, 2017; Halvorsen, 2021; Li & Zhao, 2011; Moulton & Scott, 2016). For instance, more highly educated older adults may have better access to social and economic resources and the necessary skills for self-employment. On the other hand, less educated individuals are more likely to be excluded from or face challenges in the formal labor market, which may push them into types of self-employment requiring fewer resources or knowledge (Li & Zhao, 2011). Therefore, examining how the relationship between employment transitions and health may vary depending on locality and education level is essential.

## This Study

Prior research has shown that employment is a crucial determinant of health. However, whether and how employment transitions affect health, and whether there are differences in the links between employment transitions and health by locality and education, remain underexplored. Understanding various pathways of employment transitions could offer policy and program development insights to facilitate healthy aging and longer working lives. This study makes several essential contributions to the existing literature. First, previous research on later-life self-employment has focused primarily on relatively young individuals who have not yet reached their country’s retirement age. We selected individuals aged 60 and older using the statutory definition of older adults made by the Law of PRC on the Protection of Rights and Interests of the Elderly (Chapter 1, Article 2), the same age as the mandatory retirement age. Including respondents past mandatory retirement age has implications for sustainable employment patterns and maintenance of financial and health stability later in life. Second, most self-employment research has been undertaken in Western countries. In contrast, our study incorporated the unique characteristics of the Chinese labor market, including agricultural and nonagricultural self-employment, through a longitudinal and representative Chinese dataset. Third, instead of solely focusing on the consequences of different work arrangements, this study provides a more nuanced understanding of the relationship between work and health by considering how transitions between work arrangements may affect health outcomes for older adults. Fourth, the study explored the relationships between employment transitions and health and how they vary by sociostructural factors such as hukou status, region, and education level. Finally, findings from this study could have implications for programs and practices that facilitate the quality of longer working lives and healthy aging by supporting older adults to remain in the workforce.

## Method

### Data and Sample Selection

**Figure 1.**
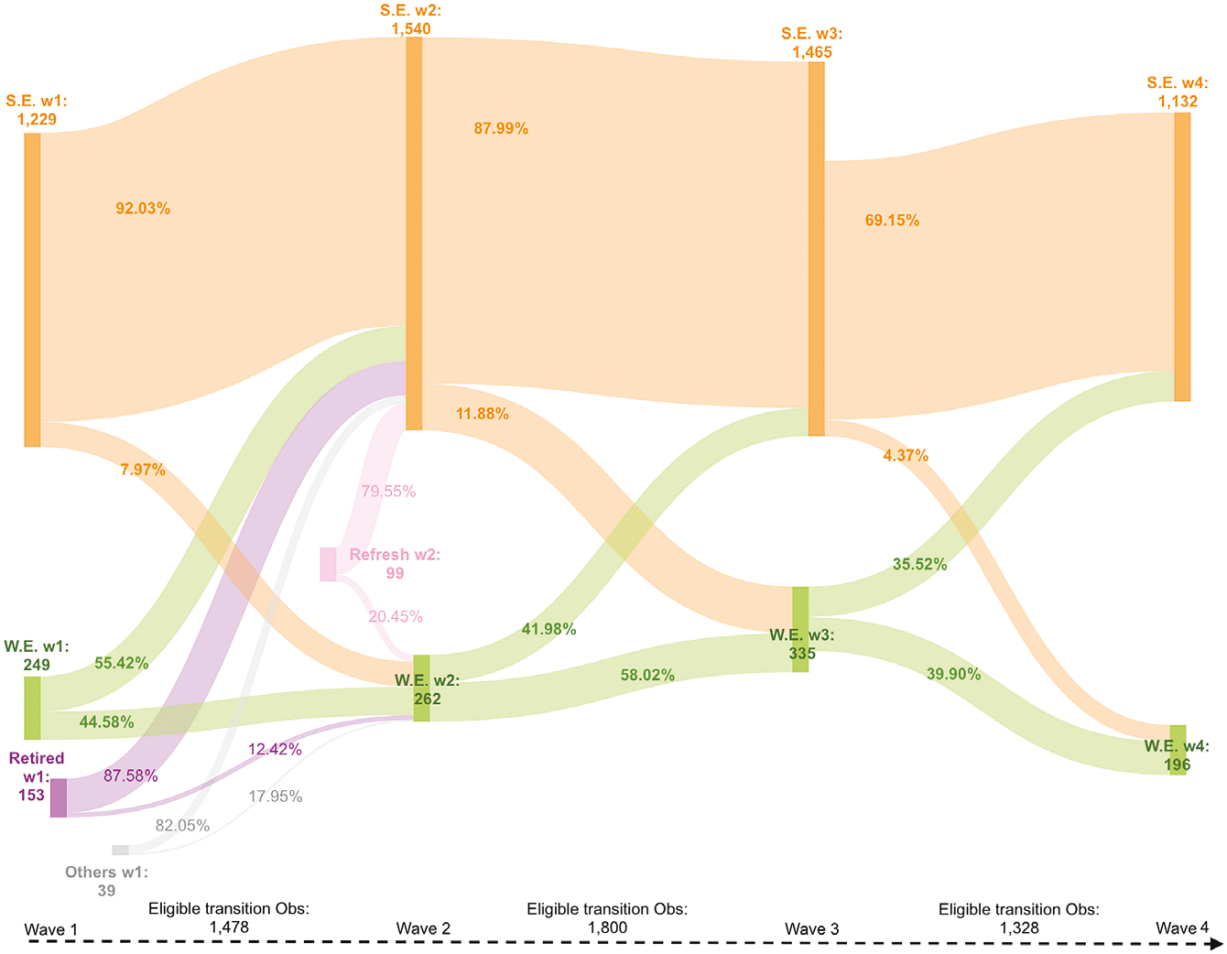
Employment transitions of all samples across waves. Unit of analysis: individual observation. This Sankey diagram shows transitions in self-employment and waged employment across waves. S.E.= self-employment. W.E.= waged employment. W1 = wave 1 (CHARLS 2011); W2 = Wave 2 (CHARLS 2013); W3 = Wave 3 (CHARLS 2015); W4 = Wave 4 (CHARLS 2018). Obs = observations.

This study analyzed four waves (2011, 2013, 2015, and 2018) of pooled data from the Harmonized China Health and Retirement Longitudinal Study (CHARLS), a nationally representative longitudinal study of individuals over 45 in China. The study sample included working respondents aged 60 and older, and the unit of analysis was the employment transition from one wave to the next (i.e., from time *t* to *t* + 1). We selected workers because this study examined employment transitions between self-employment and wage employment, requiring participants to have available employment records. This means our sample only included those who continued working during the study period or those who had previously retired or were unemployed but had re-joined the labor force in 2011. Those who were unemployed (not currently engaging in work but searching for a job in the past month) and retired (not currently engaging in work and searching for a job in the past month) were excluded from the analyses. Furthermore, to robustly estimate the transition effects, we followed Correia (2015) to ensure each individual had at least two transition observations to prevent the singleton issue that biased the estimates. Such a methodological consideration determined that our sample selection should include those participating in CHARLS for at least three consecutive waves; this also made us include a refreshed sample added in 2013 CHARLS. Figure 1 presents the overall sample characteristics of employment transitions in each wave (for the sample flows by subgroups, see Supplementary Figures 1–3).

### Measurement

#### Outcomes: physical, mental, cognitive health, and satisfaction

The study analyzed four health and well-being outcomes: cognitive, mental, and physical health and life satisfaction. Cognitive function was measured using the Telephone Interview of Cognitive Status (TICS), which has satisfactory psychometric properties in older Chinese adults (Luo et al., 2019). The TICS score uses combined data relating to episodic memory (*range*: 0–20) and other cognitive abilities (*range*: 0–10), resulting in a final variable (*range*: 0–30). Episodic memory was measured as the total score of delayed and immediate word recall, in which respondents were asked to repeat words from a 10-word list read to them immediately and after a delayed time spent answering other survey questions. Additional cognitive abilities included recognizing date, subtracting 7s from 100 (up to five times), and redrawing a previously shown picture. Depressive symptoms, used as a proxy for mental health, were measured by the sum score (*range*: 0–30) of 10 items from the Chinese version of the Center for Epidemiology Studies Depression Scale (CESD-10), which has satisfactory reliability and validity in middle-aged and older Chinese samples (Cheng & Chan, 2005). Self-rated health (1 = *very poor* to 5 = *very good*) was used to measure physical well-being and the subjective aspect of physical health. Lastly, life satisfaction (1 = *not at all satisfied* to 5 = *completely satisfied*) was included to capture subjective well-being. Sensitivity analysis in which we dichotomized self-rated health and life satisfaction revealed results consistent with our main findings; see Supplementary Table 1).

#### Predictor: employment transitions

We used the employment measures constructed by the Harmonized CHARLS (Phillips et al., 2021), in that it offers clean and ready-to-use data that specify individuals in various employment statuses, such as self-employment, waged employment, unemployed (i.e., not currently engaging in work but searching for a job in the past month), or retired (i.e., not currently engaging in work and searching for a job in the past month). The construction of the employment transitions was based on the available reported employment status (waged employment or self-employment) between two consecutive waves (*t* and *t* + 1; i.e., 2011–2013, 2013–2015, and 2015–2018). Four types of transitions were constructed: waged to self-employment (*transition to S.E.*), self-employment to waged employment (*transition from S.E.*), and those remaining in self-employment (*staying S.E.*) or waged employment (*staying W.E.*). Furthermore, as self-employment in China includes both agricultural and nonagricultural employment and the relationships examined in our analysis may differ between these categories, we differentiated agricultural from nonagricultural self-employment. Three categories of self-employment were constructed: agricultural self-employment (agricultural *S.E.*), nonagricultural self-employment (nonagricultural *S.E.*), and combined self-employment (combined *S.E.*), including both agricultural and nonagricultural self-employment. The measures of the agricultural- and nonagricultural *S.E.* were extracted from the Harmonized CHARLS data.

#### Covariates

Following previous research (Nikolova, 2019; Stephan et al., 2020), we controlled two types of time-varying confounders at baseline (time *t*) related to employment and health: demographic characteristics and health and economic factors. Demographic variables included age, age squared, hukou status, and marital status. Health and economic variables included activities of daily living (ADLs), individual and household income, and net household wealth. We used inverse hyperbolic sine (IHS) transformation to correct the skewness of income and wealth variables (Friedline et al., 2015). Time-invariant variables (e.g., gender, education, and region) were not controlled as fixed-effect models were used (Wooldridge, 2015).

### Analysis

We used a two-way fixed effect model to adjust the unobserved time-invariant individual characteristics in the panel nature of the CHARLS. We used a lagged design to estimate the associations between employment transitions (from time *t* to *t* + 1) and health (at time *t* + 1). To account for the endogeneity of self-selecting into self-employment or waged employment, we controlled for a series of time-varying demographic, health, and economic variables (e.g., age, marital status, hukou status, income, wealth, ADLs) at time *t* before each transition occurred. The results were estimated by examining the impacts of employment transitions on health and were estimated by three categories: the combined measure of agricultural and nonagricultural self-employment, agricultural self-employment, and nonagricultural self-employment. We followed the methods by Luo and Chong (2019) to explore the subgroup variations. As significant differences were observed by region, hukou, and education (see Supplementary Table 2–4), the fixed effect models were stratified by these important variables. We used multiple imputations with chained equations by creating 50 imputed data sets following White et al’s. (2011) suggestions to address missing values, particularly in income and wealth measures. The results were combined using Rubin’s rule (Rubin, 2004).

## Results

Table 1 shows baseline sample characteristics, presented by wave observations and individuals. Among all participants, nearly three-fifths (58.99%) were men, four-fifths lived in rural areas (81.19%), and most had rural hukou (93.95%). Approximately 12.87% had received a middle school education or higher. The average age of respondents was 64.66 years (*SD *= 4.35). Our sample partially reflects the features of the older Chinese working population described in previous literature: a higher proportion of rural older adults or rural hukou holders who may continue to work in the primary sector to collect or produce natural resources due to insufficient pension income and a lack of formal retirement support (Peng, 2018). Among wave-observations, approximately three-quarters (75.97%) remained self-employed across all waves; 8.58% remained in waged employment; 7.97% entered self-employment from waged employment, whereas 7.49% switched to waged employment from self-employment. Regarding the frequency of transitions between self-employment and waged employment, the results (see Supplementary Table 5) showed that 16.15% experienced one transition, 9.77% experienced two transitions, and 1.55% experienced three transitions.

**Table 1.** Sample Characteristics

Variables	Pooled wave observations (At baseline time *t*)		Individual observations (At the first entering time in CHARLS)	
	*M* (*SD*)	*N* (%)	*M* (*SD*)	*N* (%)
Demographics				
Age	66.39 (4.52)		64.66 (4.35)	
Marital status				
Not married		544 (11.81%)		196 (10.88%)
Married		4,062 (88.19%)		1,605 (89.12%)
Gender				
Women		1,869 (40.58%)		739 (41.01%)
Men		2,737 (59.42%)		1,063 (58.99%)
Region				
Rural		3,746 (81.33%)		1,463 (81.19%)
Urban		860 (18.67%)		339 (18.81%)
Activities of daily living (ADLs)	0.31 (0.79)		0.30 (0.78)	
Individual earned income (IHS)	0.11 (0.34)		0.11 (0.33)	
Household income (IHS)	0.76 (0.81)		0.81 (0.79)	
Household net wealth (IHS)	1.84 (1.55)		1.71 (1.47)	
Hukou status				
Rural		4,298 (93.31%)		1,692 (93.95%)
Urban		308 (6.69%)		109 (6.05%)
Education				
Below middle school		4,007 (87.00%)		1570 (87.13%)
Middle school and above		599 (13.00%)		232 (12.87%)
Health and well-being outcomes				
Cognitive functioning (*range*: 0–30)	13.24 (5.23)		13.84 (5.02)	
Depressive symptoms (*range*: 0–30)	8.53 (6.10)		8.93 (6.22)	
Self-rated health (*range*: 1–5)	3.06 (0.88)		2.99 (0.86)	
Life satisfaction (*range*: 1–5)	3.22 (0.74)		3.10 (0.71)	
Transition type				
Transition to S.E.	367 (7.97)		—	
Transition from S.E.	345 (7.49)		—	
Staying W.E.	395 (8.58)		—	
Staying S.E.	3499 (75.97)		—	

*Notes: M *= mean, *SD *= standard deviation. IHS *= *inverse hyperbolic sine transformation. S.E.  *= *self-employment. W.E.  *= *waged employment. Due to one individual could have multiple employment transitions across time, the sum of each type of transition can be more than 100%. For simplicity, we only present the percentages of employment transitions for wave observations.

### Two-Way Fixed Effect Results

Table 2 shows the associations between employment transitions and four health outcomes of the whole sample, with the estimates of self-employment divided into combined, agricultural, and nonagricultural self-employment. For combined self-employment (i.e., including both agricultural and nonagricultural self-employment), older people switching to or continuing in self-employment reported lower self-rated health than those remaining in waged employment (*b* = −0.19, *SE* = 0.08; *b* = −0.24, *SE* = 0.09). Older adults who transitioned from self-employment to waged employment reported lower self-rated health (*b* = −0.17, *SE* = 0.08). No significant differences in cognitive functioning, life satisfaction, and depressive symptoms were observed.

**Table 2.** Results of Two-Way Fixed Effect Models

Variables	Cognitive functioning		Depressive symptoms		Self-rated health		Life satisfaction	
	*b*	*SE*	*b*	*SE*	*b*	*SE*	*b*	*SE*
Combined S.E. (*n* = 4,606)								
Transition to S.E.	−0.30	0.40	0.57	0.45	−0.19^*^	0.08	−0.14	0.08
Transition from S.E.	−0.35	0.39	0.17	0.47	−0.17^*^	0.08	−0.04	0.08
Staying S.E.	−0.47	0.45	0.75	0.50	−0.24^*^	0.09	−0.17	0.08
Nonagricultural S.E. (*n* = 615)								
Transition to S.E.	−0.90	1.68	1.73	1.93	−0.06	0.28	−0.65^*^	0.30
Transition from S.E.	−0.09	1.17	0.69	1.42	−0.11	0.18	−0.27	0.23
Staying S.E.	−0.81	1.58	2.36	1.93	−0.60	0.31	−0.54	0.35
Agricultural S.E. (*n* = 4,209)								
Transition to S.E.	−0.20	0.43	0.40	0.48	−0.18^*^	0.09	−0.10	0.08
Transition from S.E.	−0.35	0.43	0.16	0.51	−0.16	0.09	−0.01	0.08
Staying S.E.	−0.35	0.48	0.53	0.53	−0.23^*^	0.10	−0.15	0.09

*Notes:* S.E. = self-employment. W.E. = waged employment. Reference group = *Staying W.E.* Unit of analysis: wave observation. Covariates include age and squared term of age, activities of daily living (ADL), hukou status, individual income, total household income, marital status, and total household net wealth at baseline year 2011, 2013 and 2015. Individual and year fixed effects are controlled. *SE* are heteroskedasticity-robust standard errors.

^*^ *p* < .05.

For nonagricultural self-employment specifically, older adults who switched to self-employment reported lower levels of life satisfaction (*b* = −0.65, *SE* = 0.30). Older people who remained in agricultural self-employment reported lower levels of self-rated health (*b* = −0.23, *SE* = 0.10) than those who stayed in waged employment; older adults who switched to self-employment reported lower levels of self-rated health (*b* = −0.18, *SE* = 0.09). No significant differences in cognitive functioning or depressive symptoms were observed for transitions between self-employment and waged employment.

### Subgroup Analyses

**Figure 2.**
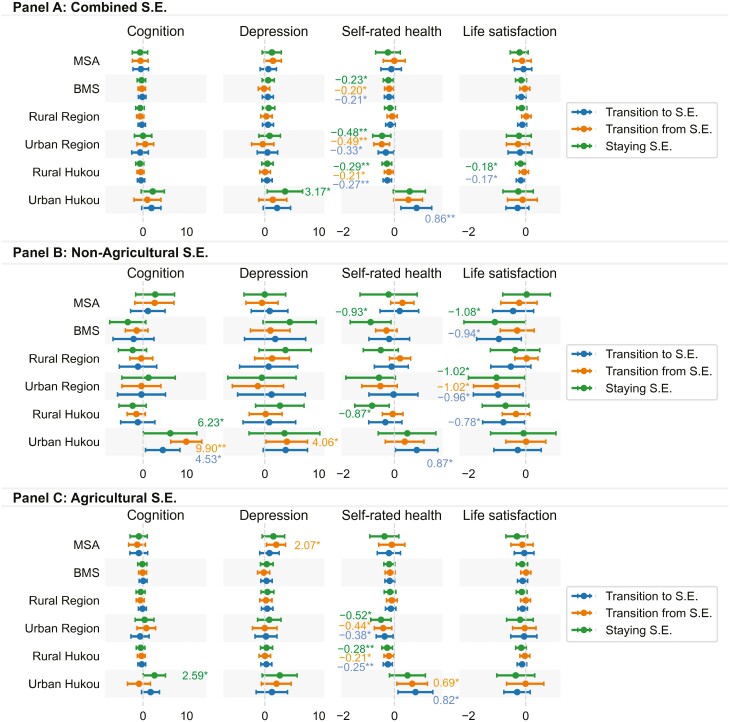
Result of two-way fixed effect models by subgroups: education, locality, and hukou. Unit of analysis: wave observation. The figures present the regression coefficients with 95% confidence intervals, stratified by education (MSA and BMS), locality (rural and urban region), and hukou (rural and urban hukou). Panels A (combined S.E.), B (nonagricultural S.E.), and C (agricultural S.E.) represent different measures of S.E. S.E. = self-employment. W.E. = waged employment. MSA = middle school and above; BMS = below middle school. Reference group = *staying W.E.* Only significant coefficients are presented. *p < .05.

Figure 2 displays the subgroup variations in the associations between self-employment transitions and outcomes by hukou, region, and education. Based on all self-employment measures, the results stratified by hukou and education revealed similar patterns. Older adults with low education levels (i.e., below middle school) or rural hukou who transitioned into self-employment or remained self-employed reported lower self-rated health and life satisfaction than those who remained in waged employment. Such adverse effects on life satisfaction were not observed among those who transitioned into or remained in agricultural self-employment. Conversely, higher self-rated health and cognition were observed among people with urban hukou who transitioned into self-employment or remained self-employed, especially for nonagricultural self-employment. Additionally, older adults with urban hukou who remained self-employed reported higher depressive symptoms. Furthermore, higher-educated older adults or those with urban hukou who transitioned from self-employment also reported higher levels of depression. Lastly, locality may also affect the relationship between self-employment and health. Those who remained self-employed or transitioned from self-employment in urban areas reported lower self-rated health and life satisfaction.

## Discussion

Our study adds new information on the impact of self-employment on health. Previous research primarily focused on Western societies and urban self-employment, treating self-employment as a static status by comparing the differences between self-employment and other types of employment. Our research investigated the longitudinal transitions between self-employment and waged employment. Further, it extends the nature of self-employment by considering urban and agricultural self-employment separately and collectively and examining their impact on health and well-being. Our findings reveal that, regardless of the types of self-employment (i.e., combined, agricultural, and nonagricultural self-employment), those who were self-employed—either remaining in or transitioning into self-employment—reported lower self-rated health than those who stayed in waged employment. Those who transitioned into nonagricultural self-employment reported lower life satisfaction than those who remained in waged employment. However, no differences were observed in cognitive functioning and depressive symptoms. Furthermore, we found heterogeneous effects of self-employment in subgroup analyses by hukou, region, and education levels. Specifically, those with rural hukou, living in urban areas, and with low education levels reported lower life satisfaction and self-rated health. However, those with urban hukou and who either stayed in or transitioned into nonagricultural self-employment had higher cognition; those who transitioned into self-employment reported higher self-rated health but worse depressive symptoms.

In contrast with previous research in Western contexts (Binder & Coad, 2013; Kautonen et al., 2017; Nikolova, 2019), we found adverse effects of self-employment on self-rated health (either transitioning into and staying in self-employment) and on life satisfaction (transitioning into self-employment). Differences in human capital and sociostructural support that either push or pull individuals into self-employment could explain such findings. For example, previous research in Germany and the U.K. has shown that individuals who switched from waged employment to self-employment were attracted by its flexibility and autonomy, which subsequently improved their perceived health and satisfaction (Binder & Coad, 2013; Kautonen et al., 2017; Nikolova, 2019). However, those pushed into self-employment because of poor socioeconomic status (e.g., lower income) or lower human capital (e.g., poor health or lower education levels) may not reap the health benefits due to uncertain and inconsistent income and experience difficulties separating their personal and professional life in self-employment (Fuchs-Schündeln, 2009; Zissimopoulos & Karoly, 2007, 2009).

In China, mandatory retirement and inadequate retirement pension provision may push older adults into self-employment, particularly those with socioeconomic disadvantages such as rural hukou and limited education. We conducted sensitivity tests comparing demographics by types of employment transitions to confirm this assumption. Supplementary Tables 6 and 7 show that older adults transitioning into or remaining in self-employment were more likely to be lower educated and have rural hukou, lower income, and poor health than those remaining in waged employment. The negative findings of self-employment using Chinese samples are consistent with previous research in Ukraine (Pham et al., 2018) and South Korea (Park et al., 2020), in that older adults engaged in self-employment due to compromised socioeconomic characteristics report a lower life satisfaction and poor self-rated health (Fuchs-Schündeln, 2009; Zissimopoulos & Karoly, 2007, 2009). Furthermore, self-employment may have adverse lingering effects on health even for those no longer self-employed, as we found that those who switched from self-employment to waged employment exhibited lower self-rated health, possibly due to their previous self-employment experiences. Our analyses of the negative impacts on health resulting from employment transitions (either transition to *S.E.* or Transition from *S.E*.) suggest that the mechanisms connecting employment and health may be complex. The negative impacts on health could be affected by the nature of self-employment (e.g., difficulties separating their personal and professional life), the transitions between *S.E.* and *W.E.* (e.g., uncertainty or income changes in the employment transition), or the motivations behind the job changes (e.g., being generative, workplace discrimination, or lack of skills; see Fuchs-Schündeln, 2009; Virtanen et al., 2008). We urge more studies to distinguish the pathways to health further through which mechanisms.

Although previous research shows that self-employment is associated with cognition and depressive symptoms, we did not find such significant associations, particularly among the overall sample. There are several plausible explanations for these insignificant results. First, as Table 1 shows, our study contained older adults predominately with lower education levels holding rural hukou and living in rural areas. Previous research has shown that education is integral for cognitive development (Zeng et al., 2022), and those with lower education may have insufficient cognitive reserves (Carret et al., 2003). Additionally, research comparing depressive symptoms between urban and rural Chinese older adults shows that the latter exhibit higher levels of depression (Wang et al., 2018). Therefore, the differences in cognition and depressive symptoms may not be observed due to these homogeneous characteristics in the study samples. Second, methodological differences may also explain the variations in study findings. Unlike previous research that simply compared the differences between self-employment and waged employment (Ahn, 2020; Chanda & Banerjee, 2022; Patel et al., 2020), we used longitudinal analysis to investigate the transitions between self-employment and waged employment. Although we found significant associations between types of employment transitions and depressive symptoms and cognition, these significant associations became muted after controlling for the individual fixed effect. This suggests that other individual and socioeconomic characteristics may capture the effects between employment transitions and mental and cognitive outcomes. Nevertheless, the current evidence on the associations between self-employment and depressive symptoms and cognition remains inconclusive as both significant (Ahn, 2020; Stephan et al., 2020) and insignificant (Caliendo et al., 2022; Parslow et al., 2004) results were reported. We encourage future researchers to examine the impact of self-employment on mental and cognitive health using varied research designs and population settings.

Our subgroup analyses by hukou and education yielded more detailed yet nuanced results between employment transitions and health, reflecting unique sociostructural features in China. Unlike the main results showing the negative impacts of self-employment on health, we found that older adults with urban hukou—who either transitioned into self-employment or remained self-employed—reported increased cognitive functioning and higher self-rated health. Such positive associations are consistent with previous research showing that favorable aspects of self-employment, such as autonomy and self-direction, can stimulate brain activity and increase perceived health (Nikolova, 2019; Stephan et al., 2020). This may be especially relevant for older adults with urban hukou, as they have more resources, economic advantages, and capacities to reap the health benefits of self-employment than rural hukou holders. However, we also observed higher depressive symptoms among older adults with urban hukou. This suggests that, although urban hukou holders are entitled to better education and welfare schemes (i.e., pension and social insurance), the nature of self-employment—longer working hours and income uncertainty—compounded with a feeling of being left out of the traditional employment economy, may impair their mental well-being (Patel et al., 2020). Furthermore, our findings also suggest that less educated people are more likely to be self-employed, consistent with previous Chinese (Li & Zhao, 2011) and Western (Caballero, 2017; Moulton & Scott, 2016) research. Previous research has indicated that, in China, older individuals with lower education levels are more likely to have informal jobs, short job tenure, and work in the agricultural sector, leading to lower pension income and savings and more discrimination in the labor market (Peng, 2018). As a result, they may be pushed into self-employment to support themselves economically in later life.

Regarding the analysis for rural–urban regions, we found that self-employment is negatively associated with life satisfaction and self-rated health among older adults living in urban areas. Due to limited job opportunities and the changing occupational structure that requires technical- and management-level jobs, China’s labor markets tend to favor younger and well-educated individuals; older adults are more likely to be steered to manual labor (Gong et al., 2015). Additionally, a significant portion of the floating population—older adults with rural hukou who migrate to or live in urban areas—are more likely to face labor market and welfare discrimination resulting from their social status (Song, 2016). The mismatch of occupational structure, discrimination against older adults with less human capital, and institutional arrangement due to the hukou system may push older adults in urban areas into self-employment, leading to poor health conditions later in life.

This study has several limitations. First, detailed information related to employment changes (e.g., reasons or motivations, either voluntarily or not) and specific types of self-employment (e.g., types of business or entrepreneurship) are unavailable in CHARLS. Therefore, the theoretical applications of push-pull factors and the mechanisms linking employment transitions and health lack the specificity that more detailed employment information would have allowed. Second, we limited our sample to working populations instead of the entire older adult sample to construct the employment transitions, with additional methodological considerations to obtain robust estimates (see Correia, 2015) by selecting respondents who participated in CHARLS for three consecutive waves. However, those who retired before or in 2011 could still join the labor force, and such “post-retirement” samples may influence the study associations. As CHARLS had no data before 2011, this study could not model this population. However, 8.5% of samples that retired in 2011 were identified. The sensitivity test (see Supplementary Table 8) showed no differences (except for gender and income) in this sample compared to the remaining sample; these significant variables were handled using fixed effect models. We also examined this working sample with excluded samples. The results (see Supplementary Table 9) showed significant differences in age, hukou, region, income, and wealth. Although these effects were considered in the fixed effect models, our findings were based on younger samples with fewer economic resources thus the applicability to women, older ages, and those with higher income or wealth may have constraints. These sample sensitivity tests suggest that our findings cannot be generalized to nonworking samples (e.g., retired, unemployed, or never worked) and participants who had sporadic instead of consistent employment status during the study period.

Third, the unique feature of CHARLS data is that it can differentiate employment by sector (i.e., agricultural and nonagricultural) for both self-employment and waged employment. This implies that individuals could have various forms of employment transitions, including the “between” job transitions (i.e., changes between *W.E.* and *S.E.*), the “within” job transitions (i.e., changes between agricultural and nonagricultural sectors within *W.E.* or *S.E.*), or the complex “between-within” job transitions (e.g., from agricultural *W.E.* to nonagricultural *S.E.*). However, due to sample size issues, determining the health impacts of the “within” and “between-within” employment transitions may not be feasible. This issue is reflected by our comparisons of self- and waged-employment when dividing it into agricultural and nonagricultural sectors. The results (see Supplementary Table 10) showed that, compared to those who stayed in nonagricultural waged employment, those who remained self-employed or transitioned into self-employment reported lower life satisfaction. These effects were nonsignificant when looking at the transition between agricultural waged employment and all types of self-employment. However, due to the limited observed sample size, some coefficients were not estimable when we differentiated waged employment into subgroups.

Fourth, the study sample included predominately older adults living in rural areas or with rural hukou. Therefore, the interpretation of employment transitions on health may be skewed to those with lower education levels or from under-resourced backgrounds. Thus, such a homogeneous sample could further influence the associations between employment transitions and health. Lastly, although we controlled time-varying variables (e.g., ADLs, income, and wealth) and used fixed effect models to capture time-invariant individual-specific characteristics (e.g., gender, education attainment), hidden or unobserved time-varying characteristics may still influence the associations between self-employment transitions and health.

Our findings have implications for programs, practices, and future research to facilitate the quality of longer working lives. As older adults’ participation in the labor force continues to rise, many societies see continued employment beyond retirement as one of the strategies to promote healthy aging and strengthen economic security in later life (Lee & Kim, 2017). However, our study suggested that older adults may be pushed into self-employment due to economic disadvantages, as the comparisons showed that those who were self-employed or transitioning into self-employment were much older and reported less income. Thus, their subsequent self-employment may lead to worse health and well-being outcomes. Therefore, policies and programs should provide economic and health support to the older population, especially those with socioeconomic disadvantages and facing more challenges in pursuing a continued working life. For instance, for rural hukou holders and those living in the urban region in particular, improving accessibility to the social welfare system, integrating the pension system, or strengthening redistribution in benefit provisions could mitigate the adverse effects of transitioning to self-employment on health. The actionable strategies include a series of policy amendments, such as hukou reform to relax welfare entitlement to residents, unified pension insurance for both urban and rural areas, and the establishment of integrated community-based services for social, health, and employment issues. For those with insufficient education, encouraging continuing education and job coaching, perhaps similar to the U.S. Senior Community Service Employment Program (Halvorsen et al., 2023), may help them find suitable jobs. Additionally, to prevent older adults from being pushed into work due to individual or structural disadvantages (e.g., workplace ageism or lack of skills), efforts related to eliminating discrimination, providing skills training, and creating age-friendly and equal-opportunity employment are crucial to achieving longer and healthier working lives, especially for older adults facing challenges in pursuing or engaging employment (Morrow-Howell et al., 2017).

Our study also offers insights for future research to further investigate the health impacts of employment transitions. First, occupational characteristics, such as industrial types, job nature, or types of business, should be considered to further differentiate waged employment and self-employment. Second, a broader range of employment transitions, such as transitions across various types of employment (e.g., *W.E.*, *S.E.*, unemployment, and retirement) or complex employment transitions (e.g., changes between agricultural and nonagricultural sectors across *W.E.* and *S.E.*) could be considered to better understand the potential health consequences of various transitional pathways. Third, this study only considers subgroup variations by education, locality, and hukou, as evidence suggests they are closely related to employment in the Chinese context. However, future research may also consider gender and age variations, as opportunities and resources in self-employment may operate differently among women and individuals in varied age groups (Zhang, 2013; Zhao & Yang, 2021), leading to differential impacts on health. Fourth, mediational pathways such as income (Kautonen et al., 2017), mental health (Nikolova, 2019), social relatedness (Augner, 2021), and perceived self-realization (Kautonen et al., 2023) may help to explain the link between employment transitions and health in later life. Lastly, emerging research has shown that individuals’ employment behavior is contextually embedded and shaped by sociostructural forces (Luo & Chong, 2019), suggesting that infrastructure factors (e.g., population density or shares of secondary and tertiary industries) that facilitate social and economic development within the community may affect the intention for self-employment. As this study only examines individual attributes, we encourage future research to consider both contextual and individual impacts on employment to provide a clearer picture of the employment transitions and the health impacts in later life.

## Supplementary Material

igae073_suppl_Supplementary_Materials

## Data Availability

This analysis uses data from the Harmonized CHARLS data set and codebook from the Gateway to Global Aging Data (Version D from June 2021). This study was not preregistered.
