# Supplementary material for: Self-Employment Transitions and Health Outcomes in Later Life: Evidence From China
Source: Innov Aging. 2024 Aug 11;8(9):igae073. doi: 10.1093/geroni/igae073 (PMC11441317; doi:10.1093/geroni/igae073)
Supplement: igae073_suppl_Supplementary_Materials [file igae073_suppl_supplementary_materials.docx]

***Innovation in Aging* Supplementary Material: Hu et al. Self-Employment Transitions and Health Outcomes in Later Life: Evidence from China.**

**Table S1.** Robustness checks for dichotomized self-rated health and life satisfaction

| Variables | Self-rated health | | Life satisfaction | |
| --- | --- | --- | --- | --- |
|  | *b* | *SE* | *b* | *SE* |
| Combined S.E. | | | | |
| Transition to S.E. | −0.08* | 0.08 | 0.01 | 0.03 |
| Transition from S.E. | −0.09* | 0.08 | 0.03 | 0.03 |
| Staying S.E. | −0.11* | 0.09 | −0.00 | 0.04 |
| Non-Agricultural S.E. | | | | |
| Transition to S.E. | −0.16 | 0.10 | −0.24* | 0.10 |
| Transition from S.E. | −0.11 | 0.07 | −0.12 | 0.07 |
| Staying S.E. | −0.21 | 0.15 | −0.30** | 0.11 |
| Agricultural S.E. | | | | |
| Transition to S.E. | −0.07* | 0.03 | 0.03 | 0.03 |
| Transition from S.E. | −0.09* | 0.03 | 0.06 | 0.03 |
| Staying S.E. | −0.10* | 0.04 | 0.02 | 0.03 |

*Note.* Unit of analysis: wave observation. Covariates include age and squared term of age, activities of daily living (ADL), hukou status, individual income, total household income, marital status, and total household net wealth at baseline year 2011, 2013, and 2015. Individual and year fixed effects are controlled. S.E.= self-employment. W.E.= waged employment. Reference group = *Staying W.E.* **p* < .05, ***p* < .01, ****p* < .001.

**Table S2.** Comparison of the analytical sample between urban and rural samples

| Variables | Urban areas | Rural areas | *t / χ^2^* |
| --- | --- | --- | --- |
|  | *M* (*SD*) or *N (%)* | *M* (*SD*) or *N (%)* |  |
| Age | 64.26 (4.36) | 64.75 (4.34) | −1.88 |
| Gender (Women) | 141(41.59) | 598 (40.87) | 0.06 |
| Men | 198(58.41) | 865 (59.13) |  |
| Hukou (Rural) | 276 (81.42) | 1416 (96.85) | 115.35*** |
| Urban | 63 (18.58) | 46 (3.15) |  |
| Education (Below middle school) | 285 (84.07) | 1,285 (87.83) | 3.47 |
| Middle school and above | 54 (15.93) | 178 (12.17) |  |
| Marital status (Not married) | 33 (9.73) | 163 (11.15) | 0.57 |
| Married | 306 (90.27) | 1,299 (88.85) |  |
| Individual earned income (IHS) | 0.19 (0.44) | 0.03 (0.19) | 4.61*** |
| Total household income (IHS) | 1.04 (0.89) | 0.76 (0.83) | 4.47*** |
| Total household net wealth (IHS) | 2.19 (1.78) | 1.88 (1.56) | 4.27*** |
| ADLs | 0.20 (0.56) | 0.32 (0.82) | −2.58* |
| Cognitive functioning (*range*: 0-30) | 14.40 (4.69) | 13.70 (5.08) | 2.16* |
| Life satisfaction (range: 1-5) | 3.14 (0.69) | 3.09 (0.71) | 1.12 |
| Depressive symptoms (*range*: 0-30) | 7.88 (5.68) | 9.18 (6.31) | −3.42** |
| Self-rated health (range: 1-5) | 3.09 (0.80) | 2.96 (0.88) | 2.38* |

*Note.* Unit of analysis: individual observation. IHS*=*inverse hyperbolic sine transformation. **p* < .05, ***p* < .01, ****p* < .001.

**Table S3.** Comparison of the analytical sample between urban hukou and rural hukou samples

| Variables | Urban hukou | Rural hukou | *t / χ^2^* |
| --- | --- | --- | --- |
|  | *M* (*SD*) or *N (%)* | *M* (*SD*) or *N (%)* |  |
| Age | 64.26 (4.36) | 64.67 (4.36) | −0.08 |
| Gender (Women) | 21(19.27) | 718 (42.43) | 22.72*** |
| Men | 88 (80.73) | 974 (57.57) |  |
| Region (Rural) | 46(42.20) | 1416 (83.69) | 115.35*** |
| Urban | 63(57.80) | 276 (16.31) |  |
| Education (Not literate) | 15 (13.76) | 592 (34.99) | 20.65*** |
| Literate | 94 (86.24) | 1,100 (65.01) |  |
| Education (Below middle school) | 69 (63.30) | 1,500 (88.65) | 58.64*** |
| Middle school and above | 40 (36.70) | 192 (11.35) |  |
| Marital status (Not married) | 7 (6.24) | 189 (11.17) | 2.38 |
| Married | 102 (93.58) | 1,503 (88.83) |  |
| Individual earned income (IHS) | 0.19 (0.44) | 0.10 (0.31) | 5.71*** |
| Total household income (IHS) | 1.04 (0.89) | 0.77 (0.77) | 8.06*** |
| Total household net wealth (IHS) | 2.19 (1.78) | 1.67 (1.45) | 3.95*** |
| ADLs | 0.20 (0.56) | 0.31 (0.79) | −1.97* |
| Cognitive functioning (*range*: 0-30) | 14.40 (4.69) | 13.69 (5.03) | 4.53*** |
| Life satisfaction (range: 1-5) | 3.14 (0.69) | 3.09 (0.71) | 1.38 |
| Depressive symptoms (*range*: 0-30) | 7.88 (5.68) | 9.12 (6.26) | −4.94*** |
| Self-rated health (range: 1-5) | 3.09 (0.80) | 2.98 (0.87) | 1.76 |

*Note.* Unit of analysis: individual observation. IHS*=*inverse hyperbolic sine transformation. **p* < .05, ***p* < .01, ****p* < .001.

**Table S4.** Comparisons of analytical sample between below middle school (BMS) and above middle school (MSA) samples

| Variables | BMS | MSA | *t / χ^2^* |
| --- | --- | --- | --- |
|  | *M* (*SD*) or *N (%)* | *M* (*SD*) or *N (%)* |  |
| Age | 64.76 (4.44) | 63.98 (3.56) | 2.57* |
| Gender (Women) | 711(45.29) | 28 (12.07) | 92.19*** |
| Men | 859 (54.71) | 204 (87.93) |  |
| Region (Rural) | 1,285(81.85) | 178 (76.72) | 2.42 |
| Urban | 285(18.15) | 54 (23.28) |  |
| Hukou (Rural) | 1,500 (95.60) | 192 (82.76) | 58.64*** |
| Urban | 69 (4.40) | 40 (17.24) |  |
| Marital status (Not married) | 187 (11.92) | 9 (3.88) | 13.47*** |
| Married | 1,382 (88.08) | 223 (96.12) |  |
| Individual earned income (IHS) | 0.09 (0.29) | 0.26 (0.51) | −7.17*** |
| Total household income (IHS) | 0.77 (0.78) | 1.13 (0.87) | −4.94*** |
| Total household net wealth (IHS) | 1.67 (1.42) | 2.01 (1.78) | −2.78** |
| ADLs | 0.32 (0.81) | 0.19 (0.52) | 2.36* |
| Cognitive functioning (*range*: 0-30) | 13.22 (4.89) | 17.99 (3.71) | −13.21*** |
| Life satisfaction (range: 1-5) | 3.08 (0.71) | 3.21 (0.68) | −2.35* |
| Depressive symptoms (*range*: 0-30) | 9.20 (6.25) | 6.97 (5.60) | 4.88*** |
| Self-rated health (range: 1-5) | 2.96 (0.85) | 3.15 (0.92) | −3.09** |

*Note.* Unit of analysis: individual observation. MSA = middle school and above; BMS = below middle school. IHS*=*inverse hyperbolic sine transformation. **p* < .05, ***p* < .01, ****p* < .001.

**Table S5.** Frequency of transitions between self-employment (S.E.) and waged employment (W.E.)

|  | Staying  S.E. | Staying W.E. | Transition between S.E. and W.E. | | |
| --- | --- | --- | --- | --- | --- |
|  |  |  | 1 time | 2 times | 3 times |
|  | *N* (%) | *N* (%) | *N* (%) | *N* (%) | *N* (%) |
| Total respondents (*N*=1802) | 1,221  (67.76%) | 86  (4.77%) | 291  (16.15%) | 176  (9.77%) | 28  (1.55%) |
| Locality |  |  |  |  |  |
| Rural region (*N*=1463) | 1,014  (69.31%) | 54  (3.69%) | 227  (15.52%) | 146  (9.98%) | 22  (1.50%) |
| Urban region (*N*=339) | 207  (61.06%) | 32  (9.44%) | 239  (70.50%) | 64  (18.88%) | 30  (8.85%) |
| Hukou |  |  |  |  |  |
| Rural hukou (*N*=1680) | 1,142  (67.98%) | 78  (4.64%) | 267  (15.89%) | 165  (9.82%) | 28  (1.67%) |
| Urban hukou (*N*=122) | 79  (64.75%) | 8  (6.56%) | 24  (19.67%) | 11  (9.02%) | 0  (0%) |
| Education |  |  |  |  |  |
| Below middle school (*N*=1570) | 1,095  (69.75%) | 67  (4.27%) | 238  (15.16%) | 147  (9.36%) | 23  (1.46%) |
| Middle school and above(*N*=232) | 126  (54.31%) | 19  (8.91%) | 53  (22.84%) | 29  (12.50%) | 5  (2.16%) |

*Note.* Unit of analysis: individual observation. S.E.*=*self-employment, W.E.*=*waged employment.

**Table S6.** Baseline characteristics by employment transition types, ANOVA tests

| Variables | Transition to S.E. (1) | Transition from S.E. (2) | Staying W.E.  (3) | Staying S.E.  (4) | *F* test | Post-hoc test |
| --- | --- | --- | --- | --- | --- | --- |
|  | *M* (*SD*) | *M* (*SD*) | *M* (*SD*) | *M* (*SD*) |  |  |
| Age | 65.54 (4.15) | 65.35 (3.91) | 64.87 (3.34) | 66.75 (4.67) | *F*=33.26*** | 4>1;4>2;4>3 |
| ADLs | 0.25 (0.66) | 0.18 (0.63) | 0.11 (0.45) | 0.34 (0.84) | *F*=14.68*** | 4>2;4>3 |
| Individual earned income (IHS) | 0.38 (0.48) | 0.08 (0.29) | 0.70 (0.62) | 0.02 (0.15) | *F*=850.33*** | 1>2;1>4;  2<3;2>4;  3>1;3>4 |
| Total household income (IHS) | 0.82 (0.78) | 0.69 (0.84) | 0.87 (0.66) | 0.76 (0.81) | *F*=1.02 | -- |
| Total household net wealth (IHS) | 1.92 (1.61) | 1.94 (1.65) | 2.11 (1.86) | 1.80 (1.50) | *F*=3.45* | 3>4 |
| Cognitive functioning | 13.90 (5.12) | 14.25 (4.92) | 14.58 (5.20) | 12.29 (5.23) | *F*=17.27*** | 4<1;4<2;4<3 |
| Life satisfaction | 3.31 (0.73) | 3.16 (0.75) | 3.25 (0.71) | 3.21 (0.74) | *F*=2.88* | -- |
| Depressive symptoms | 7.37 (5.25) | 7.62 (5.89) | 6.62 (5.15) | 8.94 (6.24) | *F*=24.03*** | 4>1;4>2;4>3 |
| Self-rated health | 3.20 (0.90) | 3.22 (0.91) | 3.29 (0.85) | 3.00 (0.88) | *F*=20.58*** | 4<1;4<2;4<3 |

*Note.* Unit of analysis: individual observation. IHS*=*inverse hyperbolic sine transformation*,* S.E.*=*self-employment, W.E.*=*waged employment. **p* < .05, ***p* < .01, ****p* < .001.

**Table S7.** Chi-square test for baseline demographic by employment transition types

| Variables | Transition to S.E. | Transition from S.E. | Staying W.E. | Staying  S.E. | *χ^2^* |
| --- | --- | --- | --- | --- | --- |
|  | *N* (%) | *N* (%) | *N* (%) | *N* (%) |  |
| Gender (Women) | 97 (26.43) | 106 (30.72) | 112 (28.35) | 1,554 (44.41) | 90.17*** |
| Men | 270 (73.57) | 239 (69.28) | 283 (71.65) | 1,945 (55.59) |  |
| Marital status (Not Married) | 37 (10.08) | 30 (8.70) | 53 (13.42) | 424 (12.12) | 5.56 |
| Married | 330 (89.92) | 315 (91.30) | 342 (86.58) | 3,075 (87.88) |  |
| Region (Rural) | 293 (79.84) | 279 (80.87) | 283 (71.65) | 2,891 (82.62) | 28.84*** |
| Urban | 74 (20.16) | 66 (19.13) | 112 (28.35) | 608 (17.38) |  |
| Hukou status (Rural) | 338 (92.10) | 324 (93.91) | 358 (90.63) | 3,278 (93.68) | 15.13* |
| Urban | 29 (7.90) | 21 (6.09) | 37 (9.37) | 221 (6.31) |  |
| Education (Below middle school) | 306 (83.38) | 281 (81.45) | 312 (78.99) | 3,108 (88.83) | 46.37*** |
| Middle school and above | 61 (16.62) | 64 (18.55) | 83 (21.01) | 391 (11.17) |  |

*Note*. Unit of analysis: individual observation. S.E.*=*self-employment*,* W.E.*=*waged employment. **p* < .05, ***p* < .01, ****p* < .001.

**Table S8.** Comparison of the analytical sample between samples without and with retirement history

| Variable | Samples with no retirement history | Samples retired in 2011 but re-joined the labor force | *t / χ^2^* |
| --- | --- | --- | --- |
|  | *M* (*SD*) or *N (%)* | *M* (*SD*) or *N (%)* |  |
| Age | 64.65 (4.35) | 64.74 (4.36) | −0.23 |
| Gender (Women) | 658 (39.90) | 72 (47.06) | 9.84** |
| Men | 991 (60.10) | 81 (52.94) |  |
| Region (Rural) | 1,345 (81.56) | 118 (77.12) | 1.81 |
| Urban | 304 (18.44) | 35 (22.88) |  |
| Hukou (Rural) | 1,552 (94.17) | 140 (91.50) | 1.76 |
| Urban | 96 (5.83) | 13 (8.50) |  |
| Education (Below middle school) | 1,437 (87.14) | 133 (86.93) | 0.01 |
| Middle school and above | 212 (12.86) | 20 (13.07) |  |
| Marital status (Not married) | 177 (10.74) | 19 (12.42) | 0.41 |
| Married | 1,471 (89.26) | 134 (87.58) |  |
| Individual earned income (IHS) | 0.12 (0.34) | 0.03 (0.19) | 3.04** |
| Total household income (IHS) | 0.81 (0.79) | 0.76 (0.83) | 0.76 |
| Total household net wealth (IHS) | 1.70 (1.47) | 1.88 (1.56) | −1.27 |
| ADLs | 0.36 (1.00) | 0.29 (0.75) | −1.07 |
| Cognitive functioning (*range*: 0-30) | 13.84 (4.96) | 13.77 (5.59) | 0.16 |
| Life satisfaction (range: 1-5) | 3.10 (0.71) | 3.05 (0.73) | 0.76 |
| Depressive symptoms (*range*: 0-30) | 8.98 (6.23) | 8.42 (6.07) | 1.02 |
| Self-rated health (range: 1-5) | 3.00 (0.86) | 2.88 (0.89) | 1.69 |

*Note.* Unit of analysis: individual observation. IHS*=*inverse hyperbolic sine transformation. **p* < .05, ***p* < .01, ****p* < .001.

**Table S9.** Comparison between excluded and study samples

| Variable | Excluded sample | Study sample | *t / χ^2^* |
| --- | --- | --- | --- |
|  | *M* (*SD*) or *N (%)* | *M* (*SD*) or *N (%)* |  |
| Age | 68.62 (6.69) | 64.66 (4.35) | 23.48*** |
| Gender (Women) | 2,856 (53.49) | 739 (41.01) | 83.98*** |
| Men | 2,483 (46.51) | 1,063 (58.99) |  |
| Region (Rural) | 2,795 (52.35) | 1,463 (81.19) | 465.40*** |
| Urban | 2,544 (47.65) | 339 (18.81) |  |
| Hukou (Rural) | 3,565 (69.14) | 1,692 (93.95) | 444.82*** |
| Urban | 1,591 (30.86) | 109 (6.05) |  |
| Education (Below middle school) | 4,242 (79.42) | 1,570 (87.13) | 52.81*** |
| Middle school and above | 1,099 (20.58) | 232 (12.87) |  |
| Marital status (Not married) | 1,274 (23.88) | 196 (10.88) | 139.06*** |
| Married | 4,061 (76.12) | 1,605 (89.12) |  |
| Individual earned income (IHS) | 0.07 (0.30) | 0.11 (0.33) | −4.94*** |
| Total household income (IHS) | 1.05 (0.97) | 0.81 (0.79) | 7.98*** |
| Total household net wealth (IHS) | 2.09 (1.68) | 1.71 (1.47) | 7.01*** |
| ADLs | 0.55 (1.22) | 0.30 (0.78) | 8.28*** |
| Cognitive functioning (*range*: 0-30) | 13.70 (5.59) | 13.84 (5.02) | −0.88 |
| Life satisfaction (range: 1-5) | 3.17 (0.74) | 3.10 (0.71) | 3.36** |
| Depressive symptoms (*range*: 0-30) | 8.71 (6.45) | 8.93(6.22) | −1.26 |
| Self-rated health (range: 1-5) | 2.86 (0.93) | 2.99 (0.86) | −5.02*** |

*Note.* Unit of analysis: individual observation. IHS*=*inverse hyperbolic sine transformation. The analyzed sample comprised respondents aged 60 and above with at least three wave observations. The excluded samples were respondents who were retired or unemployed. **p* < .05, ***p* < .01, ****p* < .001.

**Table S10.** Sensitive results of two-way fixed effect models

|  | Agricultural W.E. | | | | Non-Agricultural W.E. | | | |
| --- | --- | --- | --- | --- | --- | --- | --- | --- |
|  | Cognitive functioning | Life satisfaction | Depressive symptoms | Self-rated health | Cognitive functioning | Life satisfaction | Depressive symptoms | Self-rated health |
| **Combined S.E.** | | | | | | | | |
| Transition to S.E. | 1.05 | −0.08 | 0.11 | −0.15 | −0.75 | −0.16 | 0.57 | −0.20 |
|  | (0.90) | (0.19) | (1.09) | (0.21) | (0.53) | (0.09) | (0.55) | (0.11) |
| Transition from S.E. | 0.16 | −0.01 | −0.58 | −0.09 | −0.43 | −0.04 | 0.11 | −0.20 |
|  | (0.87) | (0.19) | (1.09) | (0.21) | (0.50) | (0.09) | (0.55) | (0.11) |
| Staying S.E. | 0.10 | −0.11 | 0.33 | −0.05 | −0.62 | −0.18 | 0.64 | −0.33** |
|  | (0.95) | (0.20) | (1.10) | (0.22) | (0.56) | (0.10) | (0.60) | (0.12) |
| **Non-Agricultural S.E.** | | | | | | | | |
| Transition to S.E. | 1.16 | −0.38 | −6.57 | −0.35 | −0.85 | −0.67* | 1.62 | 0.11 |
|  | (1.85) | (0.52) | (6.29) | (0.45) | (1.79) | (0.31) | (1.92) | (0.26) |
| Transition from S.E. |  |  |  |  | −0.11 | −0.34 | 0.48 | −0.20 |
|  |  |  |  |  | (1.21) | (0.24) | (1.50) | (0.18) |
| Staying S.E. |  |  |  |  | −0.69 | −0.62 | 1.15 | −0.72* |
|  |  |  |  |  | (1.64) | (0.36) | (1.84) | (0.30) |
| **Agricultural S.E.** | | | | | | | | |
| Transition to S.E. | 1.02 | −0.08 | 0.16 | −0.15 | −0.65 | −0.09 | 0.32 | −0.19 |
|  | (0.90) | (0.19) | (1.10) | (0.20) | (0.58) | (0.10) | (0.59) | (0.12) |
| Transition from S.E. | 0.22 | 0.01 | -0.56 | −0.11 | −0.54 | 0.01 | 0.03 | −0.17 |
|  | (0.88) | (0.19) | (1.10) | (0.21) | (0.56) | (0.10) | (0.60) | (0.12) |
| Staying S.E. | 0.05 | −0.15 | 0.02 | −0.07 | −0.46 | −0.13 | 0.39 | −0.31* |
|  | (0.96) | (0.20) | (1.11) | (0.22) | (0.63) | (0.11) | (0.65) | (0.14) |

*Note.* Unit of analysis: wave observation. Covariates include age and squared term of age, activities of daily living (ADL), hukou status, individual income, total household income, marital status, and total household net wealth at baseline year 2011, 2013, and 2015. Individual and year fixed effects are controlled. Heteroskedasticity-robust standard errors are in parentheses. S.E.= self-employment. W.E.= waged employment. Reference group = *Staying W.E.* **p* < .05, ***p* < .01, ****p* < .001.

**Table S11.** Sensitivity tests of employment transition and times (measured by waves)

| Variables | Cognitive functioning | | Depressive symptoms | | Self-rated health | | Life satisfaction | |
| --- | --- | --- | --- | --- | --- | --- | --- | --- |
|  | *b* | *SE* | *b* | *SE* | *b* | *SE* | *b* | *SE* |
| **Combined S.E.** | | | | | | | | |
| Transition to S.E.×wave 3 | 0.12 | (0.76) | 0.42 | (1.03) | 0.15 | (0.17) | 0.03 | (0.15) |
| Transition to S.E.×wave 4 | 1.46 | (0.81) | 0.37 | (1.04) | −0.12 | (0.18) | 0.12 | (0.15) |
| Transition from S.E×wave 3 | 0.37 | (0.76) | −2.16* | (0.99) | 0.21 | (0.16) | 0.03 | (0.15) |
| Transition from S.E×wave 4 | 0.35 | (0.94) | 0.14 | (1.23) | 0.02 | (0.19) | −0.08 | (0.18) |
| Staying S.E.×wave 3 | 0.37 | (0.49) | −0.45 | (0.68) | 0.11 | (0.11) | −0.10 | (0.10) |
| Staying S.E.×wave 4 | 0.24 | (0.60) | −0.02 | (0.11) | 0.51 | (0.72) | −0.05 | (0.13) |
| **Non-Agricultural S.E.** | | | | | | | | |
| Transition to S.E.×wave 3 | 5.93* | (2.69) | 2.07 | (3.91) | 1.25* | (0.58) | 0.51 | (0.59) |
| Transition to S.E.×wave 4 | −5.60 | (4.67) | −1.49 | (4.20) | 0.87 | (0.50) | −0.66* | (0.29) |
| Transition from S.E×wave 3 | −2.24 | (2.49) | 1.06 | (2.95) | −0.03 | (0.37) | −0.57 | (0.45) |
| Transition from S.E.×wave 4 | 2.89 | (3.64) | 2.58 | (4.00) | −0.21 | (0.73) | −0.54 | (0.56) |
| Staying S.E.×wave 3 | 1.95* | (0.85) | −1.39 | (1.23) | 0.39 | (0.21) | −0.06 | (0.19) |
| Staying S.E.×wave 4 | 0.88 | (1.16) | −1.09 | (1.30) | −0.05 | (0.24) | −0.15 | (0.22) |
| **Agricultural S.E.** | | | | | | | | |
| Transition to S.E.×wave 3 | −0.15 | (0.79) | 0.73 | (1.06) | 0.13 | (0.18) | −0.01 | (0.16) |
| Transition to S.E.×wave 4 | 1.57 | (0.84) | 0.71 | (1.08) | −0.09 | (0.19) | 0.13 | (0.16) |
| Transition from S.E.×wave 3 | 0.60 | (0.77) | −2.14* | (1.03) | 0.23 | (0.17) | 0.01 | (0.15) |
| Transition from S.E.×wave 4 | 0.64 | (0.99) | 0.14 | (1.26) | 0.14 | (0.20) | 0.04 | (0.18) |
| Staying S.E.×wave 3 | 0.38 | (0.49) | −0.29 | (0.69) | 0.09 | (0.11) | −0.09 | (0.10) |
| Staying S.E.×wave 4 | 0.37 | (0.61) | 0.59 | (0.74) | −0.04 | (0.13) | 0.02 | (0.11) |

*Note.* Unit of analysis: wave observation. Covariates include age and squared term of age, activities of daily living (ADL), hukou status, individual income, total household income, marital status, and total household net wealth at baseline year 2011, 2013 and 2015. Individual and year fixed effects are controlled. Heteroskedasticity-robust standard errors are in parentheses. S.E. = self-employment. W.E. = waged employment. Reference group = Transition to *S.E.*×wave 2, Transition from *S.E.*×wave 2, and Staying *S.E*.×wave 3, respectively.

**Figure S1.** Employment transitions of all samples across waves by locality (rural and urban regions)

**
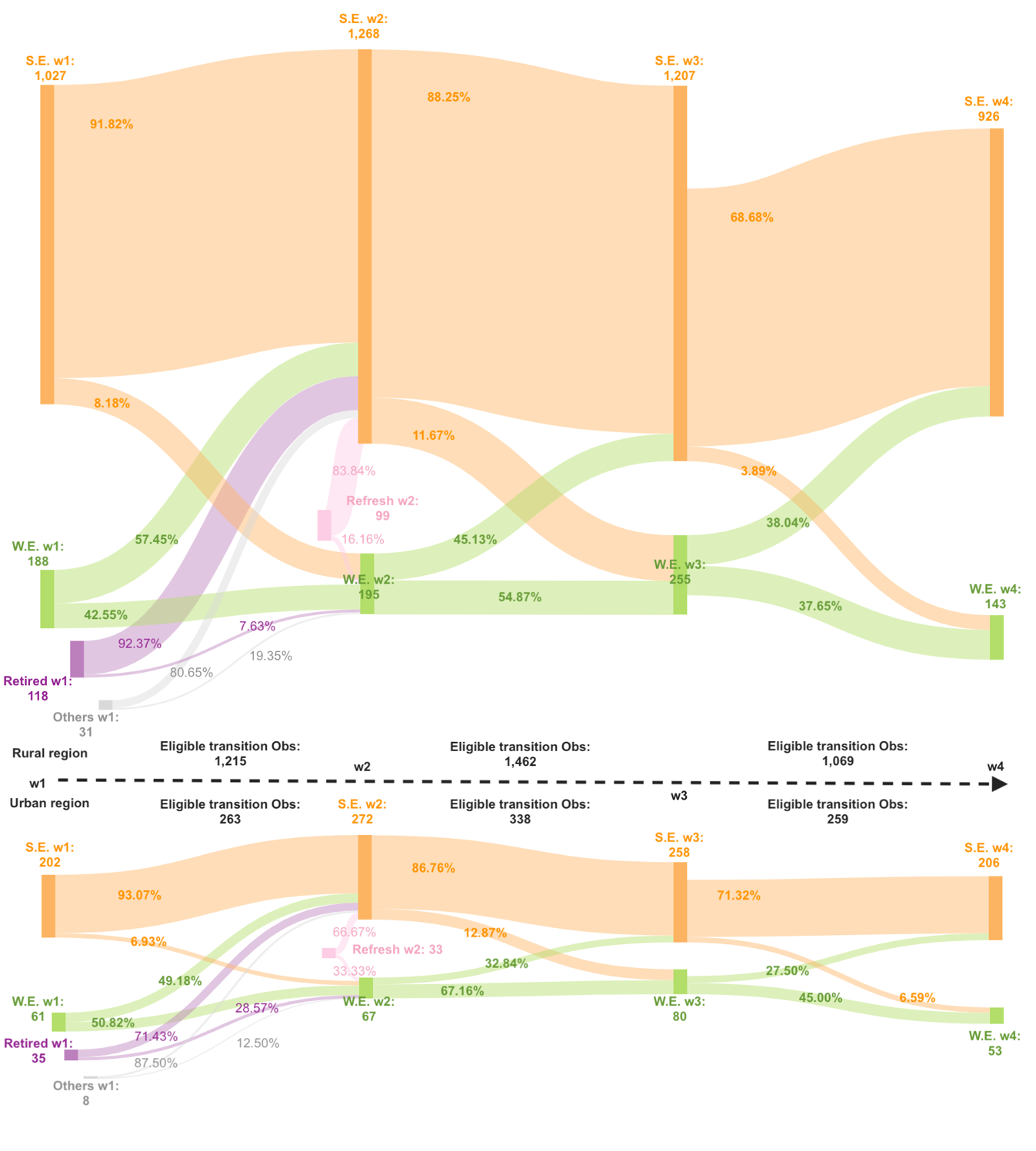
**

*Note.* Unit of analysis: individual observation. This Sankey diagram shows transitions in self-employment and waged employment across waves by locality (rural and urban regions). S.E.= self-employment. W.E.= waged employment. W1 = wave 1 (CHARLS 2011); W2 = wave 2 (CHARLS 2013); W3 = wave 3 (CHARLS 2015); W4 = wave 4 (CHARLS 2018). Obs = observations.

**Figure S2.** Employment transitions of all samples across waves by hukou (rural and urban hukou)


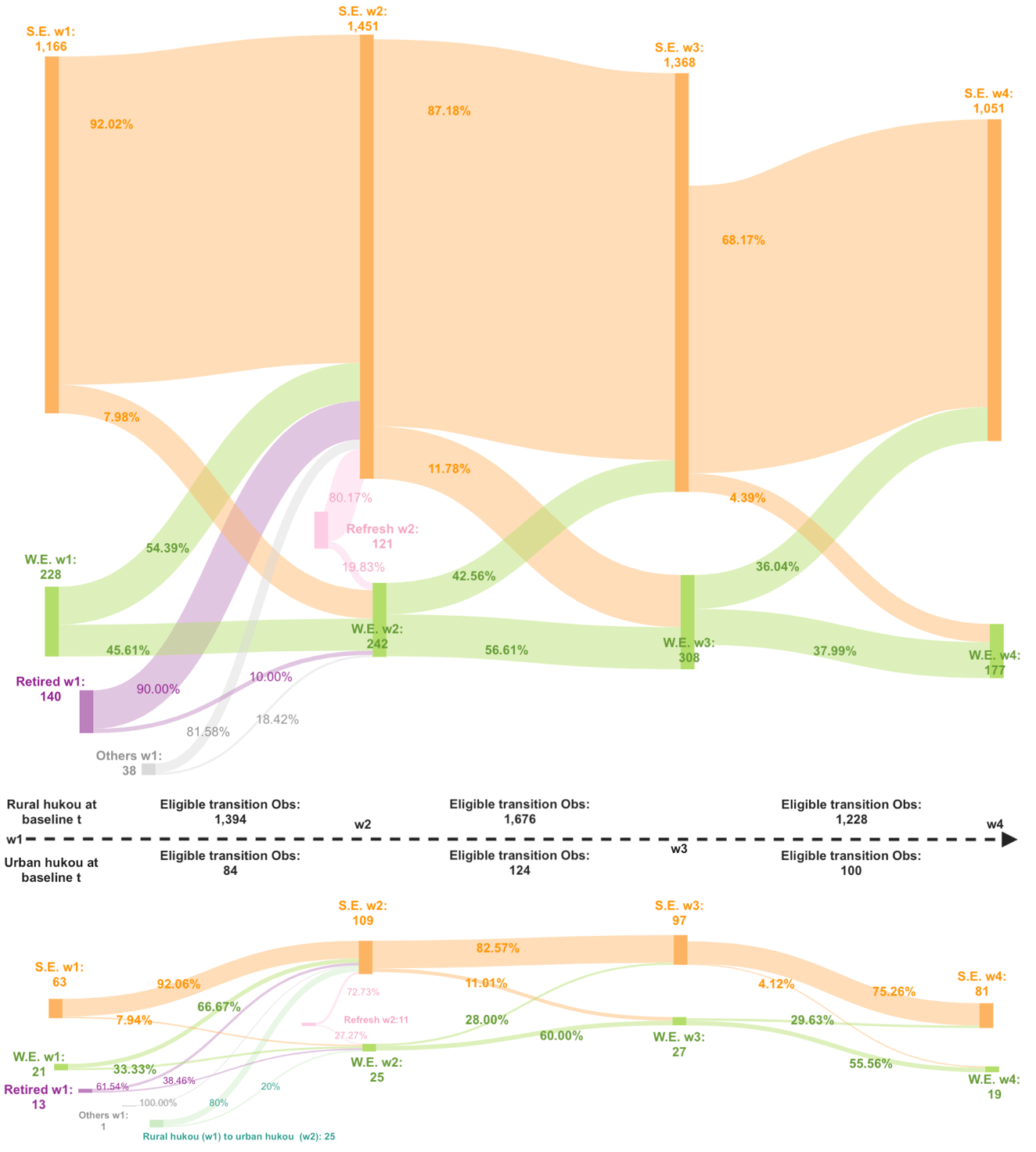


*Note.* Unit of analysis: individual observation. This Sankey diagram shows transitions in self-employment and waged employment across waves by hukou (rural and urban hukou). S.E.= self-employment. W.E.= waged employment. W1 = wave 1 (CHARLS 2011); W2 = wave 2 (CHARLS 2013); W3 = wave 3 (CHARLS 2015); W4 = wave 4 (CHARLS 2018). Obs = observations.

**Figure S3.** Employment transitions of all samples across waves by education (below middle school and middle school and above)


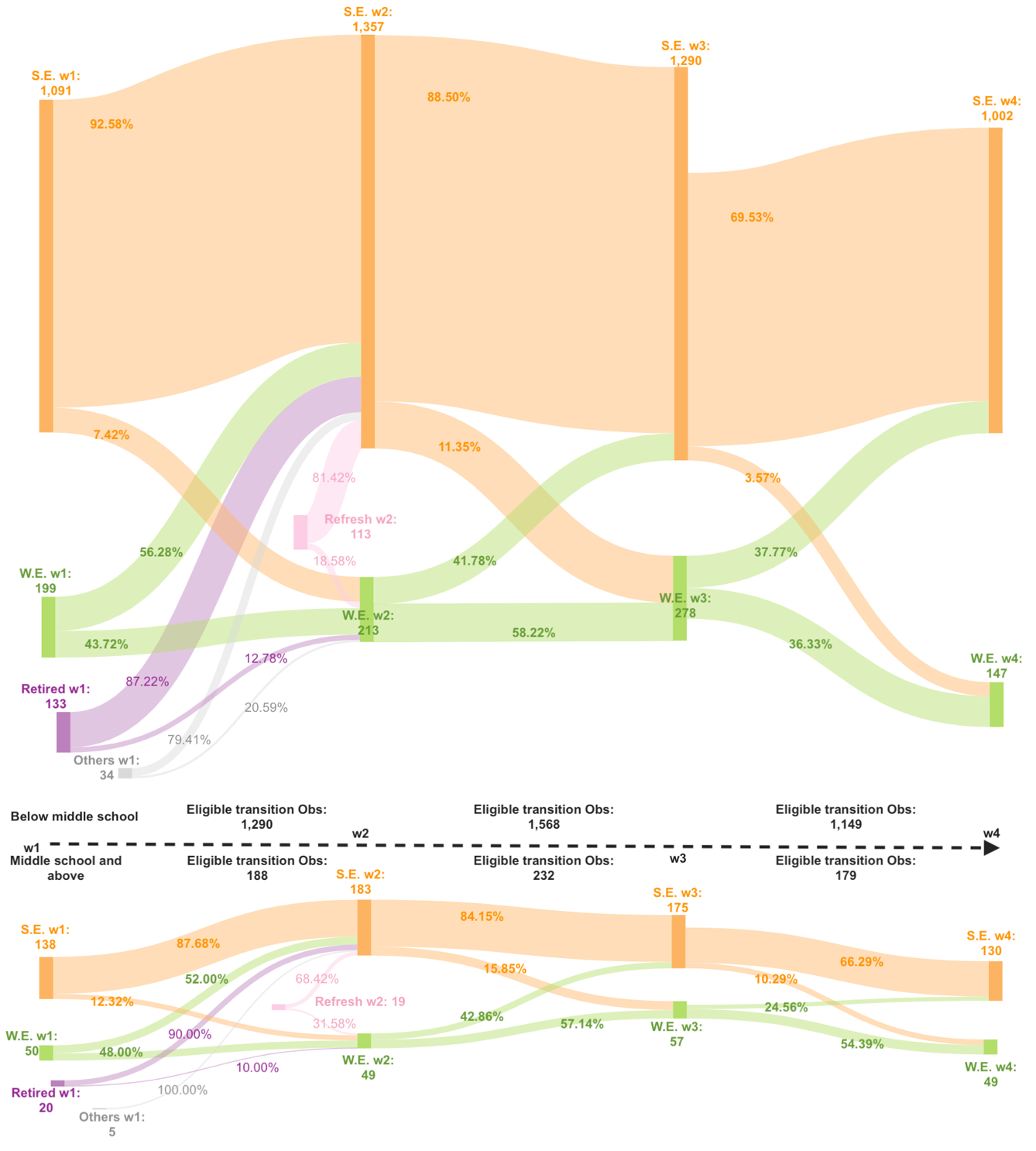


*Note.* Unit of analysis: individual observation. This Sankey diagram shows transitions in self-employment and waged employment across waves by education (below middle school and middle school and above). S.E.= self-employment. W.E.= waged employment. W1 = wave 1 (CHARLS 2011); W2 = wave 2 (CHARLS 2013); W3 = wave 3 (CHARLS 2015); W4 = wave 4 (CHARLS 2018). Obs = observations.
